# Black TiO_2_ nanobelts/g-C_3_N_4_ nanosheets Laminated Heterojunctions with Efficient Visible-Light-Driven Photocatalytic Performance

**DOI:** 10.1038/srep41978

**Published:** 2017-02-06

**Authors:** Liyan Shen, Zipeng Xing, Jinlong Zou, Zhenzi Li, Xiaoyan Wu, Yuchi Zhang, Qi Zhu, Shilin Yang, Wei Zhou

**Affiliations:** 1 Department of Environmental Science, School of Chemistry and Materials Science, Key Laboratory of Functional Inorganic Material Chemistry, Ministry of Education of the People’s Republic of China, Heilongjiang University, Harbin 150080, P. R. China; 2 Department of Epidemiology and Biostatistics, Harbin Medical University, Harbin 150080, P. R. China

## Abstract

Black TiO_2_ nanobelts/g-C_3_N_4_ nanosheets laminated heterojunctions (b-TiO_2_/g-C_3_N_4_) as visible-light-driven photocatalysts are fabricated through a simple hydrothermal-calcination process and an *in*-*situ* solid-state chemical reduction approach, followed by the mild thermal treatment (350 °C) in argon atmosphere. The prepared samples are evidently investigated by X-ray diffraction, Fourier transform infrared spectroscopy, scanning electron microscopy, transmission electron microscopy, X-ray photoelectron spectroscopy, N_2_ adsorption, and UV-visible diffuse reflectance spectroscopy, respectively. The results show that special laminated heterojunctions are formed between black TiO_2_ nanobelts and g-C_3_N_4_ nanosheets, which favor the separation of photogenerated electron-hole pairs. Furthermore, the presence of Ti^3+^ and g-C_3_N_4_ greatly enhance the absorption of visible light. The resultant b-TiO_2_/g-C_3_N_4_ materials exhibit higher photocatalytic activity than that of g-C_3_N_4_, TiO_2_, b-TiO_2_ and TiO_2_/g-C_3_N_4_ for degradation of methyl orange (95%) and hydrogen evolution (555.8 μmol h^−1 ^g^−1^) under visible light irradiation. The apparent reaction rate constant (k) of b-TiO_2_/g-C_3_N_4_ is ~9 times higher than that of pristine TiO_2_. Therefore, the high-efficient laminated heterojunction composites will have potential applications in fields of environment and energy.

The utilization of semiconductor photocatalysts for the treatment of organic pollutants^1^,^2^ and hydrogen production from water splitting^3^ has been regarded as a promising method to solve environment issue^4^ and energy crisis^5^. Among various photocatalyst materials, titanium dioxide (TiO_2_) is the most famous photocatalysts owing to its low cost, high photocatalytic activity, good stability and nontoxicity^6^,^7^,^8^. Nevertheless, the wide band-gap (about 3.2 eV for anatase) and the rapid recombination of photoinduced electron-holes are major drawbacks in its poor photocatalytic activity^9^. To date, various methods were developed to improve the visible light absorption of TiO_2_, including metal and non-metal elements doping^10^, surface sensitization^11^, semiconductor heterojunction^12^, and so on. Among them, semiconductor coupling is an efficient method to reduce the recombination of photoinduced electron-hole pairs.

Recently, graphite-like carbon nitride (g-C_3_N_4_) has been reported to be a non-toxic, stable and facile metal-free visible light photocatalyst^13^,^14^,^15^. The band-gap of g-C_3_N_4_ is ~2.7 eV, indicating a strong absorption in visible light region^16^. g-C_3_N_4_ has displayed excellent properties in photodegradation of organic contaminant^17^ and H_2_ evolution^18^. However, the key issue with the high recombination of photoinduced electron-hole pairs is still limited the photocatalytic applications of g-C_3_N_4_^19^. To resolve this problem, coupling g-C_3_N_4_ with other semiconductors has attracted much attention, which inhibits the recombination of photoinduced electron-hole pairs and thus improves catalytic performance^20^. There have been many studies on hybrids of g-C_3_N_4_ with TiO_2_. Recent studies on these composites have shown improved photocatalytic performance in dye degradation and H_2_ evolution under visible light irradiation^21^,^22^,^23^.

TiO_2_ nanobelts, have attracted great interest because of their large surface areas^24^, chemical stability^25^, and provide sufficient space for the new nucleation^26^. 2D semiconductor materials, such as g-C_3_N_4_ nanosheets, possess a unique layered structure and narrow band gap^27^, which could absorb visible light efficiently. Therefore, it is believed that 2D g-C_3_N_4_ nanosheets coupled with TiO_2_ nanobelts to form the 3D heterostructure will be a photocatalyst with superior photocatalytic activity. Moreover, the composites with high surface areas maybe produce more reaction active sites and exhibit improved photocatalytic efficiency.

To further expand the TiO_2_/g-C_3_N_4_ composite catalyst practical application, it is necessary to enhance the absorption of TiO_2_/g-C_3_N_4_ composite photocatalyst in the visible light region. During the past few decades, much effort has been devoted to make colorful TiO_2_ for better optical absorption^28^,^29^,^30^. Recently, Mao *et al*. presented black hydrogenated TiO_2_ with enhanced solar light absorption^31^. The black TiO_2_ displayed much higher photocatalytic performance over the pristine white TiO_2_, which was attributed to the higher photoinduced electron-hole pairs separation capability. The enhanced solar light absorption of the black TiO_2_ was attributed to the formed Ti^3+^ and oxygen vacancies^32^. Since then, different synthesis methods were proposed to prepare black TiO_2_, including high pressure hydrogenation, plasma assisted hydrogenation, chemical reduction, and high-temperature Al vapor reduction^33^,^34^,^35^. Therefore, the development of high photocatalytic activity based on black TiO_2_ is promising. To the best of our knowledge, up to now, few researchers report black TiO_2_ combining with g-C_3_N_4_ for pollutants degradation and hydrogen evolution under visible-light irradiation.

In this work, based on a hydrothermal-calcination method, black TiO_2_ nanobelts/g-C_3_N_4_ nanosheets laminated heterojunctions were prepared by mixing the melamine and the as-prepared TiO_2_ nanobelt, followed by an *in*-*situ* controllable solid-state reaction approach. The prepared b-TiO_2_/g-C_3_N_4_ photocatalyst with a narrow band gap exhibited excellent photocatalytic activity for methyl orange removal and hydrogen evolution under visible-light irradiation.

## Results

**Figure 1:**
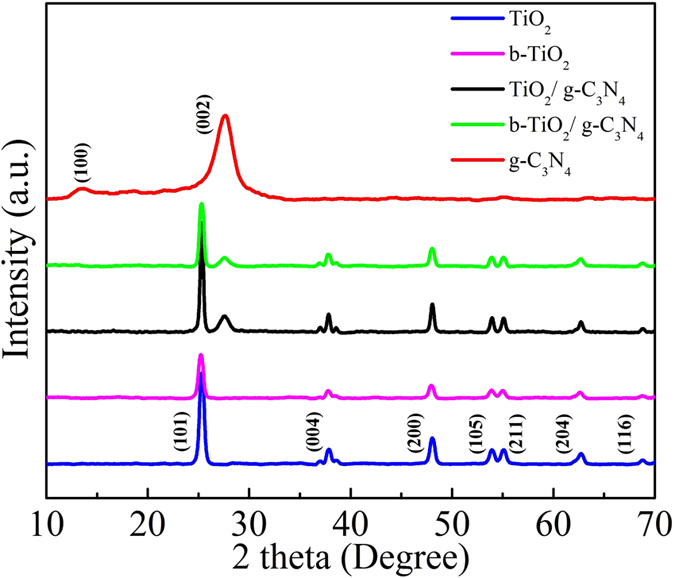
XRD patterns of TiO_2_, g-C_3_N_4_, b-TiO_2_, TiO_2_/g-C_3_N_4_, and b-TiO_2_/g-C_3_N_4_, respectively.

The samples are characterized by XRD to identify the phase composition of the samples. Figure 1 shows the XRD patterns of TiO_2_, b-TiO_2_, g-C_3_N_4_, TiO_2_/g-C_3_N_4_, b-TiO_2_/g-C_3_N_4_ composites. For pure TiO_2_, the peaks at around 25.3, 37.8, 47.9, 53.8, 55.1, 62.7, and 68.7° are ascribed to the (101), (004), (200), (105), (211), (204), and (116) crystal planes of anatase TiO_2_^26^,^36^, respectively. Moreover, the b-TiO_2_ still keeps the pristine crystal phase after the process of treatment with NaBH_4_, indicating that the crystal phase can’t be influenced by NaBH_4_. However, the XRD pattern of b-TiO_2_ shows a slightly extending characteristic peak at 25.3°, which may be ascribed to the effect of oxygen vacancies (Ov), leading the disorder-induced lattice^37^. The component g-C_3_N_4_ is characterized by two diffraction peaks at around 13.1° and 27.4° are attributed to the (100) plane and (002) plane, which correspond to in-planar structural packing and inter-planar stacking peaks of the aromatic system^38^,^39^, respectively. For the TiO_2_/g-C_3_N_4_ and b-TiO_2_/g-C_3_N_4_ samples, the XRD patterns show the characteristic diffraction peaks of both anatase and g-C_3_N_4_, indicating that the composites consisted of both anatase TiO_2_ and g-C_3_N_4_. No other characteristic peaks are found, revealing the high purity of the as-prepared samples.

**Figure 2:**
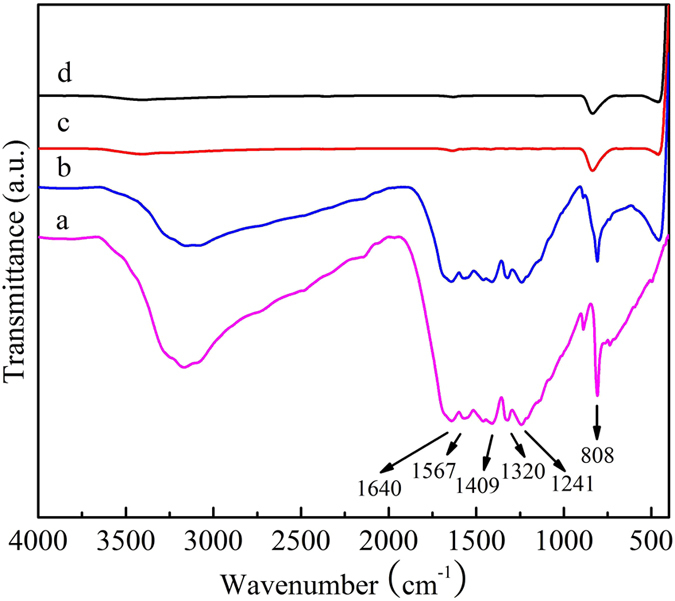
FT-IR spectra of g-C_3_N_4_ (**a**), b-TiO_2_/g-C_3_N_4_ (**b**), TiO_2_ (**c**) and b-TiO_2_ (**d**), respectively.

The FT-IR spectroscopy is applied to identify the composition of TiO_2_, b-TiO_2_, g-C_3_N_4_ and b-TiO_2_/g-C_3_N_4_ heterojunction photocatalysts, as shown in Fig. 2. For pure TiO_2_ and b-TiO_2_, the main peaks at appearing at 400–700 cm^−1^ is assigned to Ti-O-Ti and Ti-O stretching vibration modes^40^,^41^. The peaks at about 1650 and 3400–3500 cm^−1^ are corresponding to hydroxyl group and physically absorbed water on the surface of the TiO_2_^42^, respectively. In the FT-IR spectrum of g-C_3_N_4_, the absorption band at 1640 cm^−1^ can be corresponded to the C-N heterocycle stretching vibration modes^43^, while the four at 1241, 1320, 1409, and 1567 cm^−1^ to aromatic C-N stretching vibration modes^44^,^45^. The peak at 808 cm^−1^ is associated with the breathing mode of triazine units^46^. For the b-TiO_2_/g-C_3_N_4_ composite, it can be clearly seen that all the main absorption peaks of g-C_3_N_4_ and TiO_2_ appeared in b-TiO_2_/g-C_3_N_4_ composite, suggesting the presence of TiO_2_ and g-C_3_N_4_ in the as-prepared composite.

**Figure 3:**
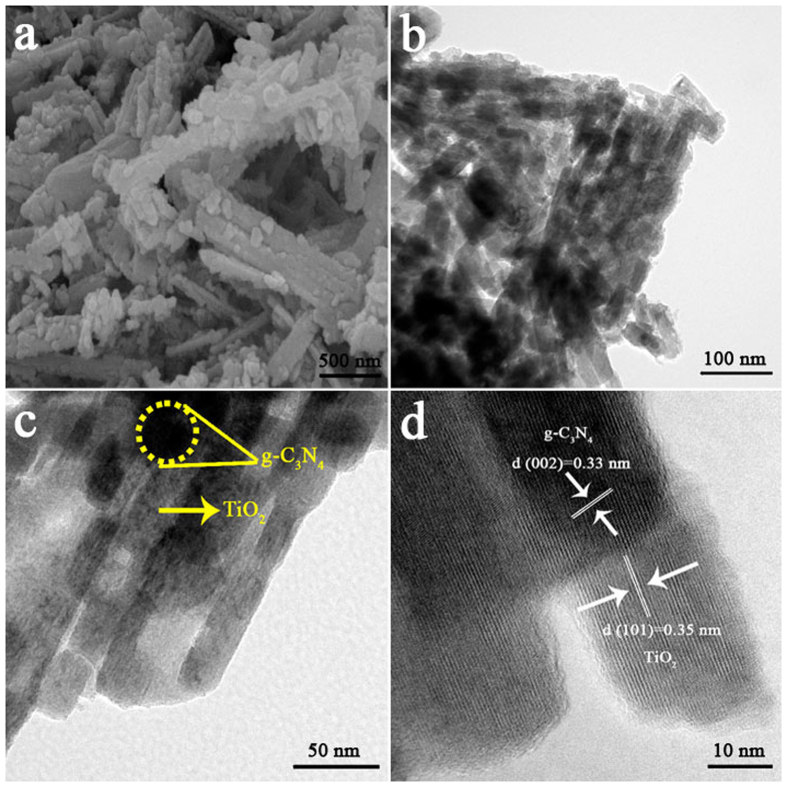
SEM and TEM images of b-TiO_2_/g-C_3_N_4_: SEM image of b-TiO_2_/g-C_3_N_4_ (**a**), TEM image of b-TiO_2_/g-C_3_N_4_ (**b**,**c**), and HRTEM image of b-TiO_2_/g-C_3_N_4_ (**d**).

The morphology and microstructure of samples are studied by SEM and TEM. Figure S1 shows the SEM image of g-C_3_N_4_, which exhibits a wrinkled sheet structure. It could be found from Figure S2 that TiO_2_ nanobelt was about 2–3 μm long, 50–200 nm wide and appeared smooth surface. The SEM image of b-TiO_2_/g-C_3_N_4_ is presented in Fig. 3a. Apparently, when compared with the pure TiO_2_ nanobelt, the surface of the b-TiO_2_/g-C_3_N_4_ composite became coarse due to the introduction of g-C_3_N_4_ nanosheet, indicating that the g-C_3_N_4_ nanosheet has been coated on the surface of TiO_2_ nanobelt and formed the laminated structure. Figure 3b and c display the TEM images of the b-TiO_2_/g-C_3_N_4_ composite, the component g-C_3_N_4_ shows a sheet shape which is coated on the TiO_2_ nanobelt. Importantly, the close contact between g-C_3_N_4_ nanosheet and TiO_2_ nanobelt is necessary for superior catalytic performance. The high-resolution TEM (HRTEM) image of composite is depicted in Fig. 3d, the lattice fringe spacing of 0.35 nm and 0.33 nm corresponded to the (101) crystal plane of TiO_2_ and (002) crystal plane of g-C_3_N_4_, respectively. The result of the HRTEM image clearly indicates the formation of special laminated heterojunctions. All these results confirmed that g-C_3_N_4_ nanosheets were successfully combined with TiO_2_ nanobelts.

**Figure 4:**
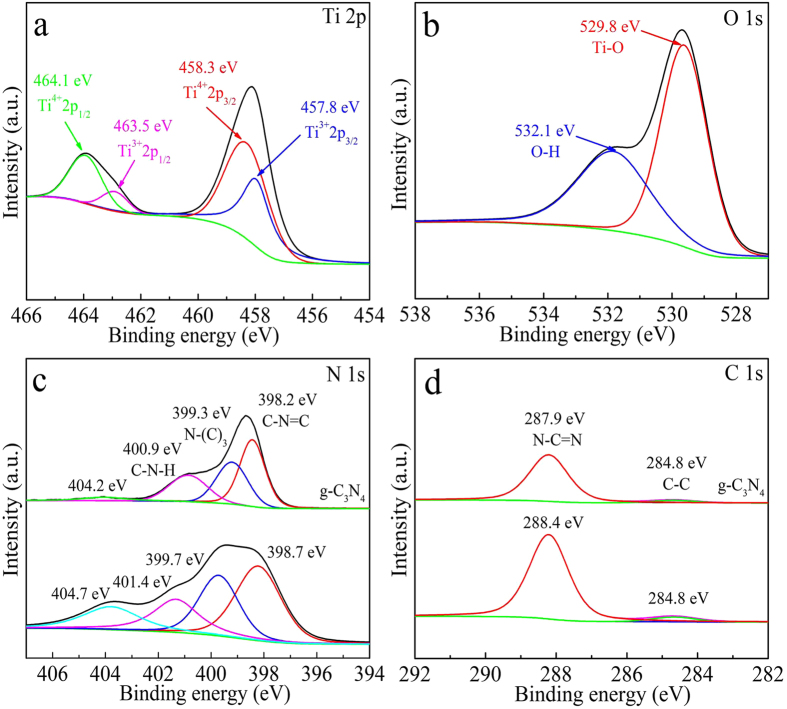
XPS spectra of Ti 2p spectra of b-TiO_2_/g-C_3_N_4_ (**a**), O 1s spectra of b-TiO_2_/g-C_3_N_4_ (**b**), N 1s spectra of g-C_3_N_4_ and b-TiO_2_/g-C_3_N_4_ (**c**), and C 1s spectra of g-C_3_N_4_ and b-TiO_2_/g-C_3_N_4_ (**d**).

In order to examine the surface chemical composition and chemical states of elements in the as-prepared g-C_3_N_4_ and b-TiO_2_/g-C_3_N_4_ sample, XPS measurements are performed. The survey XPS spectra of g-C_3_N_4_ and b-TiO_2_/g-C_3_N_4_ sample (Figure S3) reveal the presence of Ti, O, N and C elements. The results of the high-resolution XPS spectra of Ti 2p, O 1s, N 1s and C 1s of the sample are shown in Fig. 4. Figure 4a shows the Ti 2p XPS spectra of the b-TiO_2_/g-C_3_N_4_ sample, the peak located at 464.1, 463.5, 458.3 and 457.8 eV are assigned to Ti^4+^ 2p_1/2_, Ti^3+^ 2p_1/2_, Ti^4+^ 2p_3/2_ and Ti^3+^ 2p_3/2_, respectively. The Ti^3+^ species are created due to the Ti^4+^ reduction of TiO_2_ by the treatment with NaBH_4_^47^. The O 1s spectra in Fig. 4b can be fitted into two peaks, corresponding to the Ti-O bond (529.8 eV) and the -OH group (532.1 eV) on the surface of the b-TiO_2_/g-C_3_N_4_ sample^4^. Four peaks are observed in the high-resolution XPS spectrum of N 1s for g-C_3_N_4_ (Fig. 4c). The peak at 398.2 eV is assigned to sp^2^-hybridized aromatic N bound to C atoms (C = N-C), while the signal at the binding energy of 399.3 eV indicates tertiary nitrogen N-(C)_3_. The peaks at 400.9 and 404.2 eV are assigned to C-N-H groups and charging effects^6^. In N 1s XPS spectrum, compared with the g-C_3_N_4_, the peaks of g-C_3_N_4_/b-TiO_2_ shifted 0.5 eV towards higher binding energy can be attributed to the chemical environment change arising from the close interaction between g-C_3_N_4_ and TiO_2_^42^. The intensity of peaks in g-C_3_N_4_/b-TiO_2_ is higher than in pure g-C_3_N_4_ due to the existence of N defects in g-C_3_N_4_ after treatment by NaBH_4_. Figure 4d shows the XPS of g-C_3_N_4_ and b-TiO_2_/g-C_3_N_4_ in the C 1s binding energy regions. Peaks at 284.8 and 287.9 eV can be assigned to the adventitious carbon C-C and N-C = N^48^. Correspondingly, in the C 1s spectrum of the b-TiO_2_/g-C_3_N_4_ sample, the peak of N-C = N shifted 0.5 eV towards higher binding energy. The shifts of the N 1s and C 1s peaks of b-TiO_2_/g-C_3_N_4_ may be attributed to the tight contact at the interface between g-C_3_N_4_ and TiO_2_.

**Figure 5:**
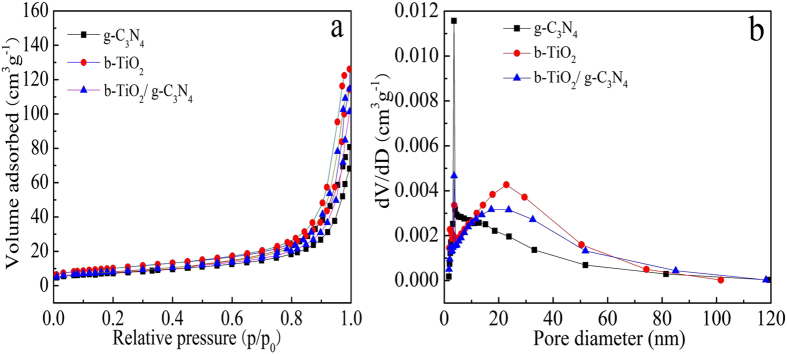
N_2_-adsorption/desorption isotherm curves (**a**) and BJH pore size distribution plots (**b**) of g-C_3_N_4_, b-TiO_2_, and b-TiO_2_/g-C_3_N_4_, respectively.

The nitrogen adsorption-desorption isotherms and the pore size distributions curves of pure g-C_3_N_4_, TiO_2_ and b-TiO_2_/g-C_3_N_4_ heterojunction photocatalyst are shown in Fig. 5a and b. It can be seen from Fig. 5a that pure TiO_2_ (36.5 m^2^/g) has a larger surface area than that of g-C_3_N_4_ (26.5 m^2^/g). Notably, the BET surface area of the b-TiO_2_/g-C_3_N_4_ composite (29.3 m^2^/g) is decreased after coupling with TiO_2_ due to the relatively low surface area of g-C_3_N_4_. Figure 5b shows the peak at 21.6 nm of b-TiO_2_/g-C_3_N_4_ is larger than the pure g-C_3_N_4_ which is the sharp peak at 3.5 nm, indicating that g-C_3_N_4_ nanosheet coated on the surface of TiO_2_ nanobelt.

**Figure 6:**
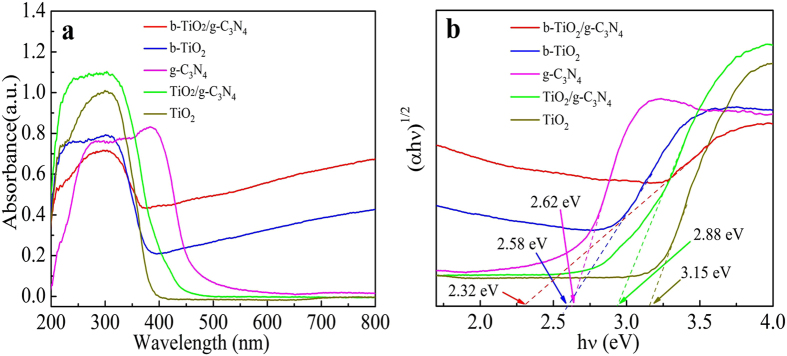
UV-visible diffuse reflectance spectra (**a**) and determination of the indirect interband transition energies (**b**) of TiO_2_, g-C_3_N_4_, TiO_2_/g-C_3_N_4_, b-TiO_2_ and b-TiO_2_/g-C_3_N_4_, respectively.

To study the light absorption ability of as-prepared samples, the UV-vis DRS analysis was performed, as shown in Fig. 6a. The absorption wavelength of g-C_3_N_4_ is up to 450 nm^39^. However, the TiO_2_ is under 390 nm which means pure TiO_2_ can only have a response to UV light^49^. After coupling with g-C_3_N_4_, the TiO_2_/g-C_3_N_4_ composite exhibits the broader absorption edge and extends to visible light region. For the b-TiO_2_, the absorption shows distinctly enhanced in the visible light region, which can be attributed to the introduction of Ti^3+^ and oxygen vacancies^32^. As can be seen clearly, the b-TiO_2_/g-C_3_N_4_ composite exhibits obvious absorption in the visible light range, due to the synergistic effect between TiO_2_, g-C_3_N_4_ and the Ti^3+^. It has been reported that Ti^3+^ and oxygen vacancies could break the selection rule for indirect transitions of TiO_2_ and improve absorption for photon energy^50^. Figure 6b shows the band gap energies of all the samples. The band gap of TiO_2_, g-C_3_N_4_, TiO_2_/g-C_3_N_4_ b-TiO_2_ and b-TiO_2_/g-C_3_N_4_ are 3.15, 2.62, 2.88, 2.58 and 2.32 eV, respectively. The narrow band gap is beneficial to improve the visible light absorption properties, so the b-TiO_2_/g-C_3_N_4_ can show an enhanced photocatalytic performance.

**Figure 7:**
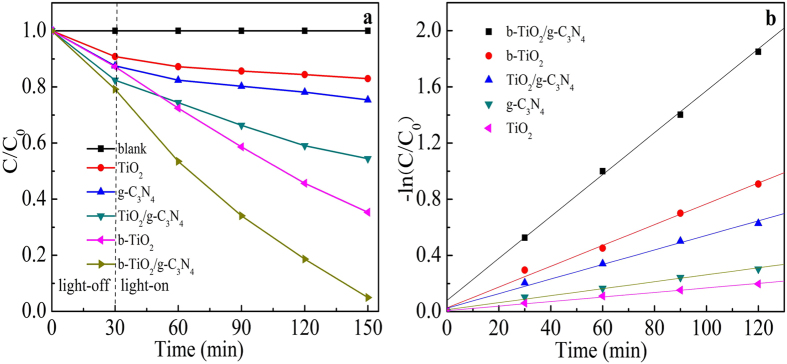
Photodegradation of MO by using different samples under visible-light irradiation (**a**), and variations of -ln(C/C_0_) versus visible-light irradiation time with different samples (**b**) (C is the corresponding degradative concentration of MO and C_0_ is initial concentration of MO).

Figure 7a shows the photocatalytic degradation of MO for different photocatalysts. The blank test demonstrates that MO could not be degraded under visible light irradiation without catalysts, and thus it can be considered that MO is stable. For pure TiO_2_ and g-C_3_N_4_, the concentration of MO is only reduced by about 17.1% and 24.6% under visible light irradiation for 120 min. The TiO_2_/g-C_3_N_4_ and b-TiO_2_ show higher photocatalytic activity, which the removal of MO is about 45.6% and 64.7%. As expected, the b-TiO_2_/g-C_3_N_4_ photocatalyst exhibits higher photocatalytic activity than other samples under visible light irradiation. The concentration of MO is reduced by about 95.1%. From Fig. 7b, the apparent reaction rate constant (k) values of TiO_2_, g-C_3_N_4_, TiO_2_/g-C_3_N_4_, b-TiO_2_ and b-TiO_2_/g-C_3_N_4_ are 0.0016, 0.0025, 0.0052, 0.0074 and 0.0153 min^−1^, respectively. Moreover, the k value of b-g-C_3_N_4_/TiO_2_ is also higher than others, which is about ~9 times higher than that of pure TiO_2_. This result suggests that introducing Ti^3+^ of black TiO_2_ and a better heterostructured combination between g-C_3_N_4_ and black TiO_2_ could promote the separation of photogenerated carriers and accelerate the electron transfer.

**Figure 8:**
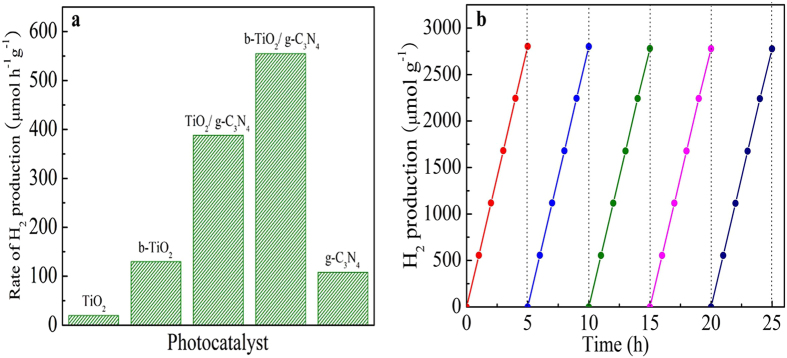
The photocatalytic H_2_ evolution of different samples (**a**) and the recyclability tests of b-TiO_2_/g-C_3_N_4_ during the photocatalytic H_2_ evolution under AM 1.5 (**b**).

The photocatalytic activity of the as-prepared samples is also evaluated for hydrogen evolution under the simulated solar light (AM 1.5) irradiation. As indicated in Fig. 8a, the pure g-C_3_N_4_ only shows a H_2_ generation rate of 108.2 μmol h^−1^ g^−1^. This is probably due to the high recombination of photoinduced electron-holes. For pure TiO_2_, very little H_2_ is produced. The hydrogen generation rate of b-TiO_2_, TiO_2_/g-C_3_N_4_ and b-TiO_2_/g-C_3_N_4_ are 130.5, 388.4 and 555.8 μmol h^−1^ g^−1^, respectively. These results indicate that the b-TiO_2_/g-C_3_N_4_ materials have the highest photocatalytic activity among the as-prepared samples, revealing that the Ti^3+^ and the heterojunction structure contribute to high photocatalytic activity. To evaluate the stability of b-TiO_2_/g-C_3_N_4_, recycling experiments were carried out on hydrogen evolution reaction for five times. As shown in Fig. 8b, the b-TiO_2_/g-C_3_N_4_ exhibits no obvious loss in hydrogen evolution activity after five cycles lasting 25 h in total, indicating the high stability of the photocatalyst.

**Figure 9:**
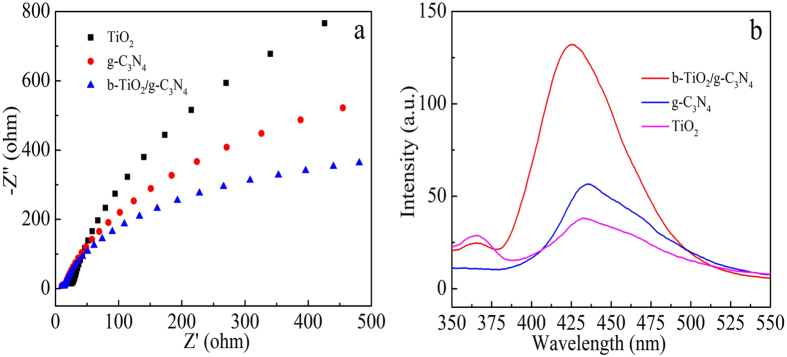
Electrochemical impedance spectra (**a**) and fluorescence intensity in 1 h (**b**) of TiO_2_, g-C_3_N_4_ and b-TiO_2_/g-C_3_N_4_, respectively.

As can be seen from Fig. 9a, the electrochemical impedance spectra (EIS) result reflects that the impedance arc radius of b-TiO_2_/g-C_3_N_4_ is smaller than that of TiO_2_ and g-C_3_N_4_ under visible light, indicating that b-TiO_2_/g-C_3_N_4_ composite demonstrates enhanced separation efficiency of the photoexcited charge carriers compared with that of pure TiO_2_ and g-C_3_N_4_. Figure 9b shows the fluorescence (FL) intensity of these samples in 1 h under Xenon lamp irradiation with a 420 nm cut-off filter. It is clearly observed that the fluorescence intensity of b-TiO_2_/g-C_3_N_4_ is the strongest than any other samples at 425 nm, indicating that the b-TiO_2_/g-C_3_N_4_ can produce the largest amount of ·OH radicals under visible light irradiation, consisting with the excellent photodegradation efficiency of MO.

**Figure 10:**
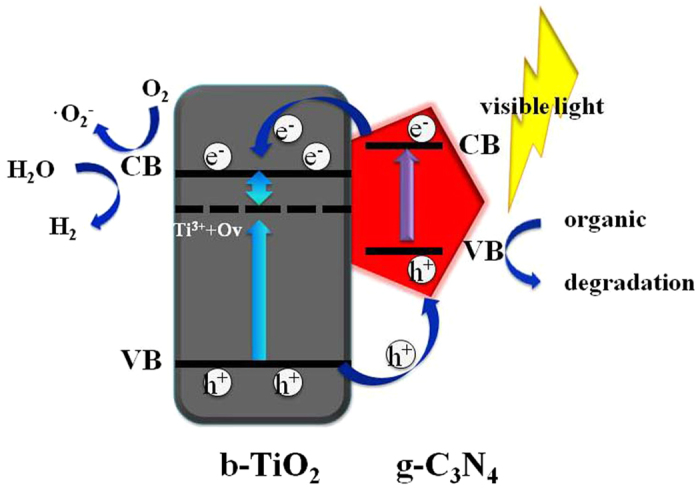
Proposed photocatalytic mechanism of b-TiO_2_/g-C_3_N_4_ composite under visible light irradiation.

On the basis of the results above, a sufficient contact interface between g-C_3_N_4_ nanosheet and TiO_2_ nanobelt is achieved. As shown in Fig. 10, the Ti^3+^ and oxygen vacancies are detected at the bottom of the TiO_2_ conduction band (CB), which can be easily narrow the bandgap of TiO_2_ nanobelt and improve the optical absorption properties^12^. When the catalyst is exposed to visible-light irradiation, g-C_3_N_4_ can produce photo-induced electron-hole pairs. The photogenerated electrons in the conduction band of g-C_3_N_4_ can transfer to the conduction band of TiO_2_^19^. Since the CB levels of TiO_2_ is more negative than the potential of O_2_/·O_2_^−^ (−0.046 eV vs. NHE at pH = 7), as a result, the electrons in CB of TiO_2_ can be trapped by dissolved oxygen to generate ·O_2_^−^ radical species. And compared with the potential of ·OH/H_2_O (2.27 eV vs. NHE at pH = 7), the remained h^+^ on the VB of g-C_3_N_4_ can not react with H_2_O to generate ·OH radicals due to the lower VB level of g-C_3_N_4_ (1.63 eV vs. NHE at pH = 7)^7^,^51^. Subsequently, the radical species ·O_2_^−^ and h^+^ can directly degrade organic pollutants. In this system, the ·OH is mainly produced by the b-TiO_2_, not g-C_3_N_4_. The separated electrons on the CB of TiO_2_ can also split water to produce H_2_^7^. The effective electron-holes separation will enhance the photocatalytic activity as compared to pure TiO_2_ and g-C_3_N_4_ due to the compact interface between the two materials.

## Conclusions

In conclusion, based on a hydrothermal-calcination method, b-TiO_2_/g-C_3_N_4_ laminated heterojunctions were prepared by mixing the melamine and the as-prepared TiO_2_ nanobelt, followed by an *in*-*situ* controllable solid-state reaction approach. The formation of a strong contact between TiO_2_ and g-C_3_N_4_ by this method greatly enhanced the separation efficiency of photoinduced electrons and holes. The narrow band gap of b-TiO_2_/g-C_3_N_4_ composite was attributed to the introduction of g-C_3_N_4_ and the Ti^3+^ species. Under visible light irradiation, b-TiO_2_/g-C_3_N_4_ composite exhibited higher photocatalytic activity than g-C_3_N_4_, TiO_2_, b-TiO_2_ and TiO_2_/g-C_3_N_4_ towards the degradation of methyl orange and hydrogen evolution. Based on this work, the b-TiO_2_/g-C_3_N_4_ composite is expected to be a highly effective visible light photocatalyst for practical applications.

## Methods

### Materials

TiO_2_ (P25) power was purchased from Deguassa Co. Ltd, Germany. Absolute ethanol (EtOH), sulfuric acid (H_2_SO_4_), and sodium hydroxide (NaOH), were purchased from Tianjin Kermel Chemical Reagent Co. LTD, China. Sodium boron hydride (NaBH_4_, 98%) was purchased from Aladdin Reagent Company, China. All reagents used in the experiments were analytical grade and employed without further purification, and the deionized (DI) water was used throughout this study.

### Preparation of TiO_2_ nanobelt

0.2 g of P25 was mixed with 40 mL of 10 M NaOH aqueous solution. The suspension was transferred to a 50 mL Teflon-lined autoclave and maintained at 180 °C for 72 h. The obtained products were washed thoroughly with deionized water and immersed in 0.1 M HCl aqueous solution for 24 h. Then the samples were immersed in a 0.02 M H_2_SO_4_ aqueous solution and maintained at 100 °C for 10 h. Finally, the products were washed with deionized water for several times and dried at 70 °C for 10 h. The sample was annealed at 600 °C for 2 h.

### Preparation of TiO_2_/g-C_3_N_4_ photocatalyst

TiO_2_/g-C_3_N_4_ photocatalyst was fabricated by calcining the mixtures of the melamine and TiO_2_ nanobelt powder. A given amount of melamine was ground with the TiO_2_ nanobelts (weight ratios of TiO_2_ nanobelt to melamine: 1:6). Finally, the mixture was calcined in a muffle furnace for 2 h at 550 °C with a heating rate of 20 °C min^−1^ in air atmosphere. For comparison, g-C_3_N_4_ was also synthesized by directly calcining melamine under air atmosphere at 550 °C for 2 h.

### Preparation of b-TiO_2_/g-C_3_N_4_ photocatalyst

**Figure 11:**
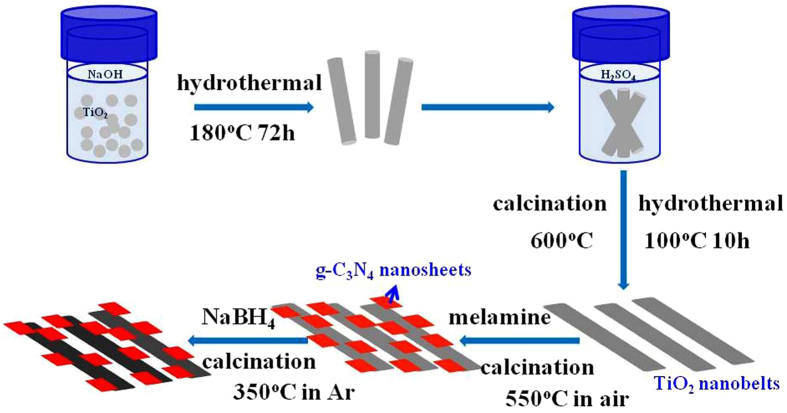
Schematic diagram for the formation of the b-TiO_2_/g-C_3_N_4_ composite.

At room temperature, 2 g of the prepared sample was mixed with 4 g of NaBH_4_ and the mixture was ground for 30 min thoroughly. Then the mixture was placed in a porcelain boat and heated in a tubular furnace for 1 h at 350 °C with a ramping rate of 5 °C min^−1^ under Ar atmosphere. After naturally cooling down to room temperature, the b-TiO_2_/g-C_3_N_4_ was obtained (Fig. 11). The obtained sample was washed with deionized water and absolute ethanol for several times. For comparison, the pure black TiO_2_ (b-TiO_2_) was also synthesized under the same condition.

The Ti^3+^ species are created due to the Ti^4+^ reduction of TiO_2_ by the treatment with NaBH_4_, so the white TiO_2_ nanobelt is turned to black^52^. NaBH_4_ reduction induces a distinctly increase in the peak intensity of Ti^3+^ and the result shows that more Ti^3+^ is formed on the surface or subsurface of b-TiO_2_, which may change the surface chemical bonding environment of TiO_2_^53^.

### Characterization

The powder X-ray diffraction (XRD) patterns were acquired on a Bruker D8 Advance diffractometer by using Cu Kα radiation (λ = 1.5406 Å). X-ray photoelectron spectroscopy (XPS) was measured on a PHI-5700 ESCA instrument with Al-*K*_*α*_ X-ray source. The Fourier transform infrared spectra (FI-IR) of the samples were collected with a PerkinElmer spectrum one system, using KBr as diluents. The morphology of the samples was observed on a field emission scanning electron microscope (FE-SEM, Hitachi S-4800). Transmission electron microscopy (TEM) was performed using a JEM-2100 electron microscope (JEOL, Japan). Surface area determination was performed by the Brunauer-Emmett-Teller (BET) method with an AUTOSORB-1 (Quantachrome Instruments) nitrogen adsorption apparatus. The UV-vis absorption spectra of the samples were measured by a UV-vis spectrophotometer (UV-2550, Shimadzu) with an integrating sphere attachment, and BaSO_4_ was used as the reference material. The ·OH radicals were detected by the fluorescence probe technique with terephthalic acid (FL-TA) on a RF-5301PC fluorescence spectrophotometer. The electrochemical impedance spectroscopy (EIS) was performed with a computer-controlled IM6e Impedance measurement unit (Zahner Elektrik, Germany).

### Photocatalytic hydrogen evolution

Photocatalytic hydrogen evolution tests were carried out in an online photocatalytic hydrogen generation system (AuLight, Beijing, CEL-SPH2N) at room temperature. The experiments were carried out by taking 50 mg of photocatalysts in a 100 mL of aqueous solution containing the 80 mL of deionized water and 20 mL of methanol used as the sacrificial reagent in closed-gas circulation reaction cell. Prior to the reaction, the system was vacuumized completely to remove O_2_ and CO_2_ dissolved in water. Then, the mixture solution was irradiated by a 300 W Xeon-lamp equipped with an AM 1.5 G filter (Oriel, USA). The hydrogen was periodically analyzed using an on-line gas chromatography with the interval of each 1 h (SP7800, TCD, molecular sieve 5 Å, N_2_ carrier, Beijing Keruida, Ltd).

### Photocatalytic degradation

The measurement of photocatalytic activity was evaluated by the degradation of methyl orange (MO) under visible light irradiation. A 300 W Xeon-lamp with a 420 nm cutoff filter. The experimental procedures were as follows: at room temperature, 30 mg of photocatalyst was added to 30 mL of 10 mg/L MO aqueous solution, which was placed at 20 cm from the light source. Before irradiation, the suspension was magnetically stirred in the dark for 30 min to ensure an adsorption-desorption equilibrium between the photocatalysts and MO. At certain intervals, the reaction solution was centrifuged to remove the particles. Finally, the concentration of MO was measured at λ = 464 nm by using a T6 UV-vis spectrophotometer.

## Additional Information

**How to cite this article:** Shen, L. *et al*. Black TiO_2_ nanobelts/g-C_3_N_4_ nanosheets Laminated Heterojunctions with Efficient Visible-Light-Driven Photocatalytic Performance. *Sci. Rep.* **7**, 41978; doi: 10.1038/srep41978 (2017).

**Publisher's note:** Springer Nature remains neutral with regard to jurisdictional claims in published maps and institutional affiliations.

## Supplementary Material

Supplementary Information
