# Supplementary material for: Black TiO2 nanobelts/g-C3N4 nanosheets Laminated Heterojunctions with Efficient Visible-Light-Driven Photocatalytic Performance
Source: Sci Rep. 2017 Feb 6;7:41978. doi: 10.1038/srep41978 (PMC5292731; doi:10.1038/srep41978)
Supplement: Supplementary Information [file srep41978-s1.doc]

## Supplementary Information

**Black TiO_2_ Nanobelts/g-C_3_N_4_ Nanosheets Laminated Heterojunctions with Efficient Visible-Light-Driven Photocatalytic Performance**

Liyan Shen^1^, Zipeng Xing^1,*^, Jinlong Zou^1^, Zhenzi Li^2^, Xiaoyan Wu^2^, Yuchi Zhang^1^, Qi Zhu^1^, Shilin Yang^1,*^, Wei Zhou^1,*^

^a^ Department of Environmental Science, School of Chemistry and Materials Science, Key Laboratory of Functional Inorganic Material Chemistry, Ministry of Education of the People’s Republic of China, Heilongjiang University, Harbin 150080, P. R. China

Tel: +86-451-8660-8616,

Fax: +86-451-8660-8240,

Email: xzplab@163.com; ysl3000@126.com; zwchem@hotmail.com

^b^ Department of Epidemiology and Biostatistics, Harbin Medical University, Harbin 150086, P. R. China


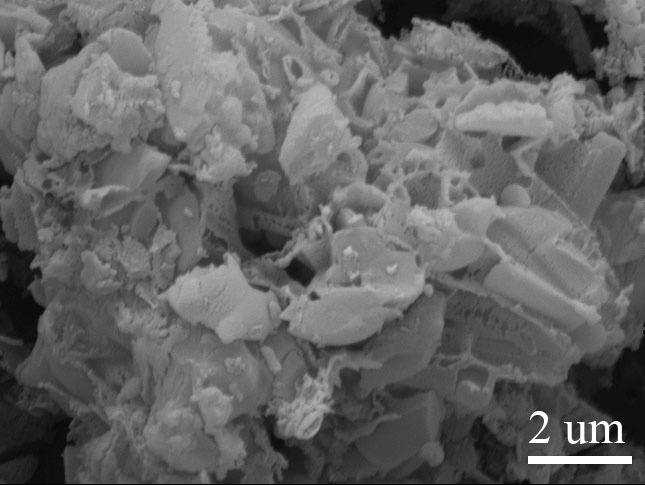


Fig. S1 SEM image of the as-prepared g-C_3_N_4_ nanosheets.


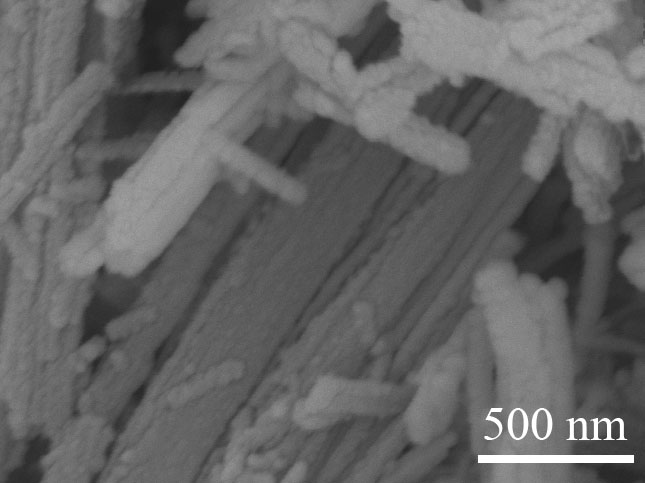


Fig. S2 Typical SEM image of the TiO_2_ nanobelts.

Fig. S3 Survey XPS spectra of b-TiO_2_/g-C_3_N_4_ and g-C_3_N_4_, respectively.


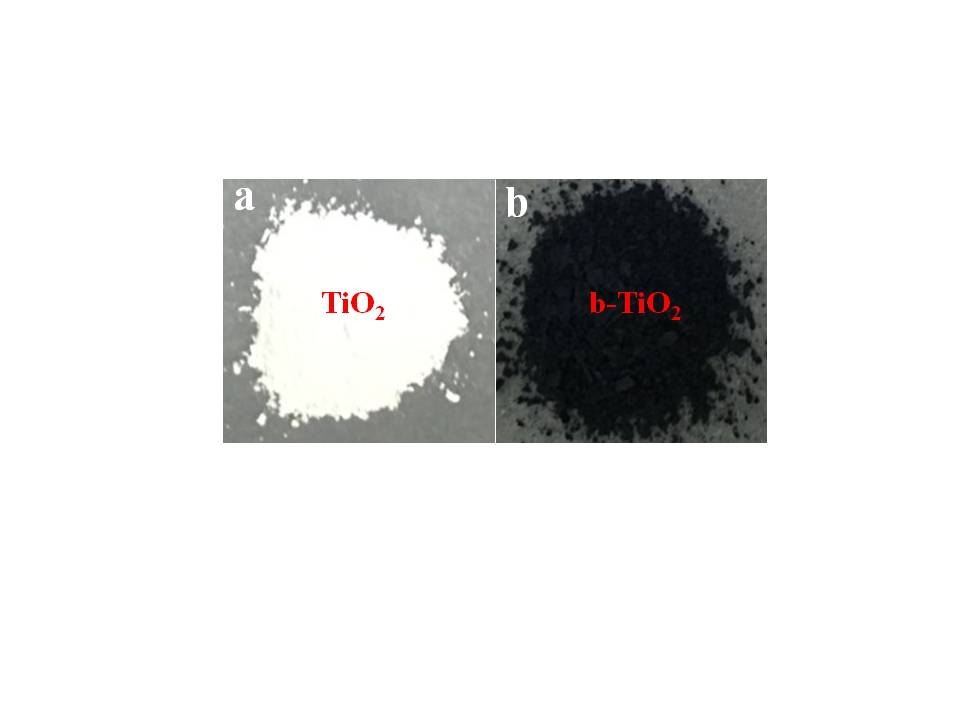


Fig. S4 Images of TiO_2_ (a) and b-TiO_2_ (b).
